# The Influence of Institutional Logics and Emotions on the Uptake of Cervical Cancer Screening: A Case Study From Xai-Xai, Mozambique

**DOI:** 10.1177/11786329231224619

**Published:** 2024-08-27

**Authors:** Gefra Fulane, Maria Major, Cesaltina Lorenzoni, Khatia Munguambe

**Affiliations:** 1 Nova School of Business and Economics, Universidade Nova de Lisboa, Lisbon, Portugal; 2 International Federation of the Red Cross and Red Crescent Societies, Geneva, Switzerland; 3 BRU-IUL, ISCTE-University Institute of Lisbon, Lisbon, Portugal; 4 Faculty of Medicine, Eduardo Mondlane University, Maputo, Mozambique; 5 Maputo Central Hospital, Maputo, Mozambique; 6 Ministry of Health, Maputo, Mozambique; 7 Manhiça Health Research Centre, Maputo, Mozambique

**Keywords:** Institutional theory, institutional logics, emotions, autonomy, cervical cancer, Mozambique

## Abstract

This study investigates the role of emotional attachment to competing institutional logics on women’s uptake of cervical cancer screening in Mozambique. Through a qualitative study conducted in Xai-Xai, Southern Mozambique, we identify 2 concurrent logics in the context of screening: preservation logic, influenced by social-cultural norms, and the prevention logic, centered around screening. Women, affected by emotions such as shame, fear, and marital subordination, often become attached to the preservation logic, which influences their values and contradicts acceptance of screening. However, some women with marital autonomy may reflect on both logics and gradually detach themselves from the preservation norms and show their intention to adopt life-saving behavior by accepting screening. It is through their emotions that women show their attachment to and detachment from competing logics, reinforcing traditional norms on the one hand, or giving them the means to adopt preventive measures on the other. The study indicates that cultural expectations, shame and the desire to preserve intimacy tie women to the logic of preservation and have a negative impact on participation in cervical cancer screening. Consequently, to improve screening uptake in Mozambique, the authorities need to adapt screening to socio-cultural and emotional factors, empower women, and effectively engage communities.

## Background

Cervical cancer is a significant global health issue, causing numerous deaths each year, particularly in low- and middle- income countries where access to preventive measures like screening is limited.^1^ In 2020, there were an estimation of 604 000 new cases of and 342 000 deaths from cervical cancer.^1^ Caused primarily by an infection with human papillomavirus (HPV), cervical cancer can be reduce by up to 80% if routine screening and timely treatment are available.^2^ To fight against cervical cancer, the Ministry of Health of Mozambique launched the National Cervical Cancer Prevention and Treatment Program in 2009.^3^ In Mozambique, where there were 5325 new cases of cervical cancer in 2020,^4^ the screening program follows a “screen-and-treat approach” utilizing visual inspection with acetic acid (VIA) for screening and cryotherapy for the treatment of pre-cancerous lesions.^5^

In accordance with national guidelines, cervical cancer screening is provided free of charge in healthcare facilities and is primarily aimed at women between the ages of 30 and 55 (referred herein as target women).^3,5^ Screening has been the predominant prevention strategy for women within this age range in Mozambique as the vaccination program was introduced to target 10-year-old girls.^6^ When screening results identify low-grade lesions (below 75%), immediate cryotherapy treatment is administered.^5^ For high-grade lesions (above 75%), women are referred to higher-level healthcare facilities for specialized treatment.^5^

Despite national efforts, only 3.5% of target are estimated to be screened every year in Mozambique.^6^ In many African contexts, barriers like limited access, lack of awareness, and low education levels have been pointed to hinder cervical cancer screening uptake.^7^ In Kenya, fear, uncertainty, poor knowledge, and cost act as primary factors affecting screening participation.^8^ Similar findings have been observed in Latin America, where barriers include limited access to quality services, high screening costs, discomfort and lack of privacy during pelvic examinations, self-negligence, fear, and anxiety over results.^9^ Hispanic women in a separate study cited cultural embarrassment, fear, and fatalism associated with cancer diagnosis.^10^ Moreover, in European countries, namely in England emotional factors such as pain, embarrassment, and discomfort were identified as barriers,^12^ while in Serbia, barriers included insufficient knowledge, limited access to curative services, gender role limitations, and inadequate health education in the community, schools, and family.^12^

Existing literature on screening barriers primarily focuses on individual and organizational factors, as well as the role of service providers, in limiting screening availability, access, and use. However, the effectiveness of a cervical cancer screening program relies on collaboration among various actors, holding different rationalities and reasoning. Cultural patterns, particularly the patriarchal system that is prevalent in Southern Mozambique,^13^ can significantly influence women’s attitudes and behaviors, potentially affecting their decision to accept preventive measures. Studies on screening barriers often lack a comprehensive understanding of women’s perspectives, including their perceptions of the importance of prevention, and how these intersect with deeply ingrained beliefs, values, and norms that are passed down through generations. Additionally, these studies overlook the connections between these cultural bonds and their intricate manifestation through complex emotions.

To understand further the role that these ingrained beliefs, values and cultural principles play in the process of women’s acceptance of screening in Mozambique we draw on the concept of institutional logics, understood as “*the socially constructed, historical patterns of cultural symbols and material practices, including assumptions, values, and beliefs, by which individuals and organizations provide meaning to their daily activity, organize time and space, and reproduce their lives and experiences*.”^14^ Initially focused on societal-level institutional orders like family, religion, state, market, professions, and corporations, the concept of institutional logics has evolved, broadening its scope to include analysis at individual, organizational, and field levels. The institutional logics perspective assumes that institutions operate across those multiple levels of analysis.^14^ Therefore, institutional logics effectively understands individuals by considering the broader social and cultural context influencing their thoughts and actions.^14^ Logics encompass constructed beliefs, rules, and assumptions that shape decision-making and choices, providing a framework for reasoning and influencing actors’ lives, identities, goals, and actions.^14^ They emerge from the collective meanings that are shared among individuals, exerting an influence on their actions and being shaped by those actions.^15^ Logics are embraced, perpetuated, and modified by individuals.^15^

In the institutional logics perspective, actors’ interests, identities, and values are shaped by social, cultural, and political contexts, resulting in coexistence of multiple and often conflicting belief systems, known as institutional complexity.^14^ In this complex environment, individuals often struggle to interpret and respond to the incompatible demands imposed by different institutional logics.^16^

Empirical studies reveal that when facing competing institutional logics, individuals employ rational interpretation to manage and sustain their coexistence, using co-option to combine elements from different logics,^17^ or by strategically blending professional and policy-driven logics to enhance their position.^18^ Although important, these studies focus primarily on rationality and cognitive interpretations and neglect the role of emotions and the unconscious in guiding individuals’ behaviors and actions. Logics are anchored in strong emotions; thus, emotions are integral to institutional logics, shaping individuals’ identities and commitments.^19,20^

Defined as *“complex, embodied but socially constructed structures of knowledge, feeling, and ethical reasoning that guide and constrain the social action and interaction that underpins institutions”*,^21^ emotions are complex social constructions performing a signaling function, indicating *“how we feel, how we wish we felt, how we try to feel, how we classify feelings, and how we express them”*.^22^ Primary emotions, such as happiness, sadness, anger, and fear, are innate and universally experienced, while secondary emotions are influenced by cultural norms and societal factor and include guilt, shame, pride, and empowerment.^22^

This research aims to contribute to the literature on barriers to cervical cancer screening by shedding light on the underlying beliefs, values, and emotions that influence women’s decision-making. It is important for policymakers and providers to recognize that simply making screening services available is not sufficient; understanding and addressing the barriers and the underlying factors that influence women’s willingness to undergo screening is crucial. This paper questions: “How do women’s emotional attachment and detachment to competing institutional logics influence the acceptance of cervical cancer screening in Mozambique?”

## Methodology

Given that institutional logics are best understood qualitatively,^23^ this study employs qualitative research techniques to explore how women in Mozambique perceive, navigate, and respond to competing logics in relation to cervical cancer screening.^24^ Qualitative methods provide in-depth and detailed information while considering the specific social and cultural contexts of the study participants.^25^ Unlike quantitative methods, qualitative approaches are flexible, facilitate fluid interactions during data collection, involve a smaller number of participants who are selected purposively, and the analysis relies on subjective interpretation through coding and categorization rather than objective verification.^24,26^

An exploratory case study approach is utilized throughout the study to gain a comprehensive understanding of how women emotionally interpret and make sense of these logics, and how this impacts the contemporary issue of cervical cancer screening in Mozambique.^25,27^ The case study method is advised for addressing research questions concerning “how” and “why,” particularly when the investigator has limited control over the events under examination. This approach is especially suitable when the research is focused on a contemporary phenomenon within its authentic real-life context.^25^ This research meets these conditions, wherein the unit of analysis (“the case”) is defined as the uptake of cervical cancer screening in Xai-Xai, Mozambique, along with an exploration of the impact of women’s emotions and reasoning in this uptake process.^25^ The research process followed the steps prescribed in case study research literature, namely: (1) designing and planning the study; (2) preparing to collect evidence; (3) collecting evidence; (4) analyzing evidence; and (5) reporting the case.^25^ These steps were carried out interactively, not in a rigid sequence, and the first author, a native of the study site, was responsible for their development.

### Research context

The empirical investigation of this paper was undertaken in Xai-Xai, Southern Mozambique, which has a population of approximately 115 752 inhabitants, distributed into 4 administrative posts. Gender inequalities are particularly accentuated in the South, and they influence patterns of disease interpretation and health care seeking.^28^ A context where the patriarchal culture and masculine social order are exceptionally strong, the position of male figures as rulers of families is commonly taken for granted.^29^ This is a reality that women accept and reproduce by associating themselves to private roles and matters related to domestic activities and procreation.^29^ Throughout the socialization, women are taught to preserve these and other social rules and cultural bonds, which condemns, for instance, the public verbalization and visualization of sexuality.^14,29^ We call this the “preservation logic.” Women who do not follow the preservation logic are considered immoral, and this threatens womanhood.^13,29^

The Ministry of Health in Mozambique has been implementing a cervical cancer screening program within the context described above. This program operates under principles and practices grounded in biomedical evidence that early detection and treatment of pre-cancerous lesions can prevent cervical cancer.^6,30^ These practices and principles are part of what we call “prevention logic.” To ensure the success of the screening program, women in the target group are expected to visit a primary-level healthcare facility, assume a childbirth position, and allow a healthcare professional to visually examine their genital area, applying a cotton swab with acetic acid to the cervix and observing any changes that may indicate the presence of low or high-grade lesions.^6,30^ Table 1 summarize the main characteristics on which identities, goals, values and decision-making capacities are expected when women are guided by the preservation logic or by the prevention logic.

**Table 1.** Example of how women are likely to understand their identity, values, goals, and decision-making capacity, if guided by preservation logic or by prevention logic.

Categories	Preservation logic	Prevention logic
Women identity	An adult female person who is mature enough to take care of her family, house, husband and safeguard marriage.	An adult female person, with enough maturity to make rational choices.
Women goals	To respect her husband, safeguard family and preserve the family and community rules.	Avoidance of circumstances that can lead to disease onset.
Women values	Community and family-based norms and beliefs, including cultural explanations of disease causation, prevention and treatment.	Scientific medicine and its explanations of disease causation, prevention and treatment.
Decision-making capacity	Women depend on her husband or other family members to make decisions on their life and health.	Women capable of making rational and effective decisions about their life and health.

Xai-Xai is particularly suitable because the goal to reach overall acceptance of cervical cancer screening goes side by side with enduring cultural values, often unquestionable, which influence people’s actions and reactions.^28,31^ In Xai-Xai, cervical cancer screening program was introduced to target the 4 administrative areas (“posts”): Sede Administrative Post, Praia Administrative Post, Inhamissa Administrative Post, and Patrice Lumumba Administrative Post. Data collection was focused on the most populated administrative posts: Sede, Patrice Lumumba, and Inhamissa. These areas are served by 3 health centres capable of providing cervical cancer screening: “Xai-Xai Health Centre” in Sede, “Patrice Lumumba Health Centre” in Patrice Lumumba, and “Marien Ngoabi Health Centre” in Inhamissa.

### Data collection and participants

The study consisted of 2 phases. During the first phase (December 2017 to May 2018), efforts were made to submit and obtain ethical approval for the research protocol by the Bioethics Institutional Committee (“Comité Institucional de Bioética em Saúde da Faculdade de Medicina/ Hospital Central de Maputo”). Subsequently, the protocol was presented in local health services, in the 3 administrative posts where data was gathered, as well as the corresponding health centres. Access to the communities and health centres was made possible with the support and facilitation of local authorities, health service managers, and nurses, who helped to obtain verbal informed consent from study participants. During this process, we obtained also various documents, reports, and guidelines pertaining to cervical cancer screening in Xai-Xai.

To explore initial community’s knowledge, perceptions, and practices regarding cervical cancer and screening to inform the refinement of the research question and interviews topics, data was gathered through focus group discussions, and observations. Focus group discussions were conducted in the main square of the 3 administrative posts, in 3 different sessions of approximately 20 participants in each session (Table 2). These participants included women as well as local leaders, who are trusted figures within the community. The duration of each of these discussions was approximately 120 minutes. The discussions were facilitated by the first author, a community health worker, and a note taker. They followed a structured guide (see appendix 1) that covered topics such as perceptions around sexuality and womanhood, knowledge about reproductive health and cervical cancer, and practices and beliefs related to screening. Both Portuguese and the local Tsonga language (Changana) were used in the facilitation. Observations were also performed by the first author. Superior approval for our study allowed close collaboration with assigned nurses at each health centre. We obtained verbal consent from both nurses and patients before conducting observations, and we ensured that patients were informed about the study’s objectives. Following this, we intermittently entered consultation rooms. Observations consisted of assessing the consultation area and screening facilities to grasp the patient-nurse behavior and the overall environment, equipment, and record-keeping. No observation of the actual screening practice was conducted. The data gathered during the initial phase revealed a heightened emotional connection to aspects related to the preservation of women’s bodies, sexuality, and intimacy.

**Table 2.** Summary of qualitative data sources.

Data collection phases	Technique	Participants
First phase (December 2017 to May 2018)	Documental review	Not applicable
	Focus group discussions	59 participants (local leaders, target women and practitioners)
	Observations	Not applicable
Second phase (June 2018 to July 2018)	Semi-structured interviews	44 interviews: 35 target women, 3 nurses, 2 managers, 4 family members.
	Informal conversations	18 conversations: 10 target women, 2 nurses, 1 manager, 5 family members.

Building upon the insights gathered from the first phase, we prepared the second phase of data collection, which took place from June 2018 to July 2018. The objective of this phase was to understand how women navigate and make sense of both the preventive aspect of screening and the cultural significance of preserving their cultural bonds, and how these factors influence their acceptance of screening. Following the procedures proposed in literature,^25,32^ we selectively chose participants who were considered relevant to the study and could offer valuable insights. These participants willingly agreed to take part in the study and provided their informed consent in written form (see appendix 2). Informed consents were subsequently archived as physical and digital copies. Semi-structured interview guides were developed for target women (see appendix 3), nurses (see appendix 4), health managers (see appendix 4), and family members (see appendix 5). The guides covered various topics, including socio-demographic information, daily routines, perceptions of womanhood, health and illness, healthcare-seeking practices, access to healthcare (geographic, financial, and cultural), knowledge about HPV infection and cervical cancer, and experiences and interpretations of the screening process.

Interviews to target women were conducted in a quiet corner of each health centre to establish a comfortable and conducive environment for open and honest exchange. Nurses informed women of the study during routine reproductive health consultations and invited those willing to contribute to join the first author in the interview corner. At the inception of each interview an explanation of the study’s objective and the participants’ right to withdraw or decline certain questions was provided. The first author was careful to build trust and empathy with study participants, and holding a naïve attitude helped to immerse into local structures and to explore experiences and perceptions. Interviews with nurses and managers took place at the health centres, while family members were interviewed in their own homes. Each interview lasted approximately 40 minutes and was conducted in both Portuguese and Changana. Semi-structured interviews were conducted with 35 target women, 3 nurses, 2 managers, and 4 family members, amounting to a total of 44 interviews (Table 2).

To gather additional insights from individuals who were not included in the formal interviews or did not consent to participate, informal conversations were conducted. These conversations occurred spontaneously in various locations such as the health centres patio and waiting rooms, as well as in participants’ homes. In total, the first author engaged in conversations with 10 target women, 2 nurses, 1 manager, and 5 family members (Table 2).

Interviews and focus group discussions were recorded for accurate documentation, while insights gathered from observations and informal conversations were carefully documented in a dedicated notebook. During both phases of data collection, the first author transcribed the gathered information word-for-word and translated the narratives from Changana to Portuguese and then into English. Other authors verified the Portuguese-English translation. Personally identifiable information was removed, and fictitious names were assigned to the study participants to ensure confidentiality. The data was stored securely on an online platform accessible only to the authors of this study.

### Data analysis

We engaged rigorously with data since the collection period and since then we went into an “endlessly creative and interpretive process.”^32^ We started by immersing ourselves in the data by reading all transcripts and translations, notes, and additional materials to identify relevant information that could respond to the research question to explore and comprehend the individual feelings and sense-making processes regarding screening. Inspired by the Gioia et al methodology,^33^ which provides a systematic framework to analyze qualitative data, the analysis followed a bottom-up process, starting with the raw data and gradually identifying patterns, themes, and insights. In this process, 2 sequential stages of analysis were performed: firstly, common themes and patterns were identified (first-order analysis); secondly, initial categories were aggregated into more meaningful and smaller number of categories following interaction with our theoretical frame (second-order analysis).^33^ In so doing, we were able to structure and code information into first-order concepts, second-order themes, and inherent dimensions.

To ensure rigor and reliability, systematic checks were implemented at various stages of the analysis. This involved cross-referencing the original audio, transcripts and data, and examining multiple perspectives, and comparing findings across different participants.^26,33^ The analysis also employed an interactive approach, allowing for ongoing engagement with the data during and after the data collection process.^26,33^ By embracing an inductive approach, the analysis aimed to capture the richness and complexity of participants’ experiences and perspectives.^33^ It allowed for new insights to emerge from the data, enabling a deeper understanding of individual emotions, thoughts, and sense-making processes related to screening. The systematic checks and interactive approach ensured that the analysis was comprehensive and rigorous, enhancing the trustworthiness of the findings.^33^

First-order concepts: during data collection and after reading the materials, we identified the main narratives that could be associated to women emotional interpretation and reaction to cervical cancer screening. Keeping the faithful originality of terms coming from informants’ narratives, we gathered different descriptions while the number of categories were coming up. We started to do an exercise to compare the emerging categories to find general similarities and differences. As myriad descriptions were identified, we decided to group together concepts that seemed to follow the same behavior, to induce patterns that showed women performing and defending their own institutional logic from one side or having the ability to be reflexive and adopt elements of the new logic from another.

Second-order themes: with different concepts described, we generated the second-order themes using axial coding. At this stage, we decided to shape the previous concepts with authors’ expertise and strong inputs from literature on institutional logics and emotions. We asked ourselves if the emerged concepts could generate themes that help explaining the complexity of women’s interpretation of logics existing in the context of cervical cancer screening in Mozambique. Using evidence on institutional logicsand gender and health care seeking in Mozambique, we started to discover that apparently social and moral emotions were either constraining or enabling women’s identities, goals, values and decisions in important matters pretraining the acceptance of cervical cancer screening. We conceptualized emotions as state of feeling that can be shorter- and longer- term experienced, including approval, support the endurance of social bonds.

**Figure 1.**
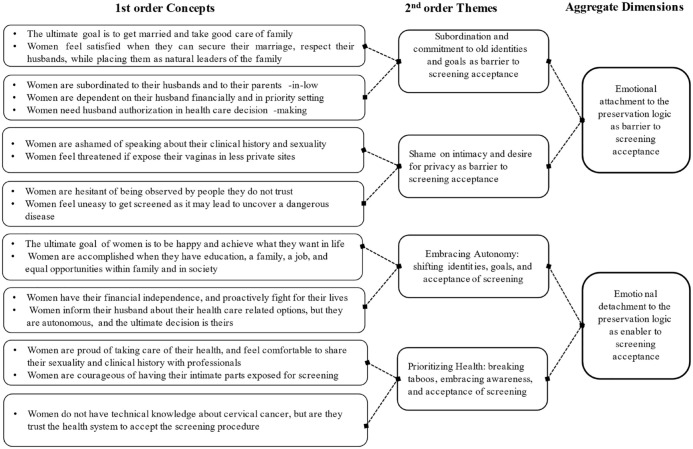
Data structure.

Aggregate dimensions: if in the previous phases, our engagement with the theoretical realm was limited, at this stage, a profound understanding of literature on inhabited institutional logics and actors’ management of competing logics were fundamental to discover theoretical paths that would help responding to our research question and build our theoretical framework. Looking over the previously identified concepts and themes with inputs from authors,^14,19,33^ we generated 2 aggregate dimensions, namely (1): Emotions and the constraining effect of institutional logics; and (2) Emotions and the enabling effect of institutional logics. The dimensions demonstrate how through emotions institutional logics constrain and enable women from undertaking cervical cancer screening in Xai-Xai. Our findings detail these dimensions and include direct quotations from participants’ narratives. Figure 1 describes the data structure process in a graphic and visual way.

## Findings

By analyzing the interplay between institutional logics and emotions, the study sheds light on the mechanisms underlying women’s acceptance of cervical cancer screening in Xai-Xai. It demonstrates how emotional attachment or detachment, influenced by institutional logics, can either constrain or enable women’s decision-making processes regarding screening.

### Emotional attachment to the preservation logic as barriers to screening acceptance

#### Subordination and commitment to old identities and goals as barrier to screening acceptance

Some participants in the study exhibited strong emotional attachments to societal expectations and cultural norms that impose limitations on their roles as women. These individuals believed that being a woman meant adhering to certain predetermined identities and goals. According to their understanding, a woman should grow up, get married, and prioritize being loyal to her husband. Additionally, “she is expected to excel in domestic responsibilities, such as house cleaning, cooking, and childcare” (Ter, 33 years old, target woman). Their perception of a “true woman” includes waking up early to fulfill these duties, not only for their immediate family “but also for their husband’s extended family” (Ju, 26 years old, target woman; Vi, 20 years old, target woman). In terms of behavior and dressing, findings show that women are expected to demonstrate respect, follow family norms, and pass these norms onto their children. “She should know her limits, and the fact that there are things she can do” (Cre, 20 years old, target woman). Moreover, their personal goals are shaped by societal and familial expectations. For instance, some women expressed a desire to continue their education and pursue employment opportunities. However, they felt obligated to prioritize their husband’s wishes, leading them to abandon their own aspirations: “I wanted to continue my studies and get a job, but I knew my husband would not approve that; he says he can afford everything; and I have to accept that” (Hel, 36 years old, target woman).

The prevailing authority in decision-making within families in Xai-Xai appears to rest primarily with the husbands. Regardless of whether the women work or not “the head of the house is always the man” (Del, 25 years old, target woman). Women often yield to their husband’s wishes, even if it means compromising their own aspirations. For instance, one participant who owned a hairdressing salon stated that she had to comply with her husband’s decision to prevent her from working: “I have a hairdressing saloon, but when my husband doesn’t want me to go there and work, I have to listen to him. I don’t do things he doesn’t want me to do” (Ter, 33 years old, target woman). In situations where the husband is absent, the influence and authority over the household decision-making are often assumed by the husband’s parents and relatives. This is particularly evident when the husband is an immigrant in the neighboring country South Africa, leaving the widowed mothers-in-law in charge (eg, 27 years old, target woman). This sense of dependence and lack of decision-making authority extends to important aspects of women’s lives, including their health. Health care professionals face difficulties in challenging long-standing norms and convincing women to seek care and undergo cervical cancer screening. Many women decline screening due to the fear that their husbands would not grant permission. It is crucial for health care professionals to approach the conversation in a way that emphasizes the importance of women’s health and wellbeing without jeopardizing their marriages: “Most of them do not accept being screened because their husband would not give permission to that. We have to put the conversation in a way that the women understand that by accepting to be screened, we do not intend to destroy her marriage, we just want her to care about her health and her life” (Ang, 31 years old, nurse).

These accounts highlight that women’s emotional attachments to traditional gender roles and societal expectations can create barriers to accepting cervical cancer screening, as they prioritize subordination to old identities and goals over their own health and well-being. This highlights the influence of patriarchal authority, which hinders women’s autonomy and decision-making power regarding their health.

#### Shame on intimacy and desire for privacy as barrier to screening acceptance

Women express reluctance when it comes to discussing their intimate lives and often keep such matters private. They only feel comfortable discussing these topics with a select few individuals they trust, such as their partners: “when I speak about it [sex] it is with my boyfriend. I don’t trust friends or anyone else” (Cel, 21 years old, target woman). Voluntarily exposing their genitals during the screening is also a challenge for many women. The conditions in the screening rooms and privacy elements play a crucial role in their decision-making. We noticed through observations that there was only a curtain separating the observation bed where women lay from the desk where the consultation began. The presence of companions, like husbands, in the consultation room creates discomfort as their voices can be overheard due to divided spaces “Some people come to the consultation room with companion such as husband and we can hear the male voice from the other side while we are being observed, because the place of consultation and the place of observation are divided by a cloth curtain” (Van, 39 years old, target woman). Older women feel embarrassed being observed by younger professionals, perceiving them as grandchildren: “they think, you are like my granddaughter; it is not acceptable to ask me to open my legs. What a shame—they complain” (Ang, 31 years old, nurse). The gender of health professional also plays a role, with some women preferring to be examined by female professionals they can rely on, as they harbor concerns about the intentions of male professionals.

Furthermore, many women lack a proper understanding of the screening procedure itself. This lack of knowledge leads to suspicions that the process could potentially harm their sensitive reproductive organs, dissuading them from participating. “They once asked me if I wanted to be observed. I denied it. I did not want because other women who had left the consulting room had whispered to me ‘you have no idea what they are doing in there: seeing our vaginas.’ This is a hidden place; and I do not understand what exactly they would do to me” (A woman from focus group discussion). However, after receiving initial counseling and becoming better informed, some women realize that the procedure is relatively straightforward: “I was afraid because I didn’t know what I was going to do; I wasn’t informed. But after being there and getting initial counsels, I realized it could be easy” (Ter, 33 years old, target woman). Some associate screening with other painful medical tests, like finger pricks for blood samples, which adds to their apprehension: “Yes. I had the chance to do it once, but I didn’t have the courage. Myself, I am unable to do a simple malaria test. It hurts, you know. I’m afraid of itching” (Del, 25 years old, target woman). There is also a belief among some women that screening may uncover a dangerous disease like cervical cancer.

Taken together, these findings highlight the influence of shame, privacy concerns, limited trust, lack of knowledge, and misconceptions as barriers that impede women’s acceptance of cervical cancer screening.

### Emotional detachment to the preservation logic as enablers to screening acceptance

#### Embracing autonomy: Shifting identities, goals, and acceptance of screening

Despite adhering to traditional social norms and maintaining stable marriages, some participants expressed a deep admiration for values such as independence, courage, and determination when discussing the meaning of womanhood. These emotional attachments shape their understanding of women’s identities and goals. True women, according to their perspective, possess the qualities of strength, independence, emancipation, and decisiveness. Being a woman “is to know how to have a work in order to not depend on someone to survive. It is also to know how to fight for what you truly need to be happy in life” (Pau, 30 years old, target woman). “I try everything with my husband, even those tasks that seem to be male” (Dul, 35 years old, target woman).

Some women actively strive for autonomy and agency in making decisions about their lives. For example, Pau, a married mother of 2, not only runs her own small business but also attends night school to obtain a degree. She greatly admires other women who work in various fields, highlighting their autonomy and independence. She thinks that “nowadays, there are much more women who work as housekeepers, in restaurants and bars, in gas stations, as mechanics in garages, women who spend nights working; and I admire these women so much for their autonomy” (Pau, 32 years old, target woman). Within their households, women take an active role in decision-making regarding household matters and health issues. The power dynamics are characterized by a sense of emancipation, as expressed by Dul, a married mother of 3, who believes in the absence of a hierarchical structure and emphasizes mutual submission between partners. “There are no bosses. We all have the right to speak. I have to be submissive to my husband, and he has to be submissive to me as well” (Dul, 35 years old, target woman). When it comes to important health decisions, women inform their husbands, not seeking permission but rather providing information, and ultimately retain the final say.

This demonstrates the transformative power of autonomy in shaping women’s identities, goals, and their willingness to engage in screening processes.

#### Prioritizing health: Breaking taboos, embracing awareness, and acceptance of screening

Women who display confidence in their decisions and actions recognize the significance of discussing their clinical history and sexual behavior as a means to understand the risks associated with cervical cancer. They value open dialogs about sexual health with both healthcare professionals and their families, including their husbands. “I feel fine to have a dialog about intimate health with health professionals and with my family, including my husband. The more I dialog, the more I learn” (Dul, 35 years old, target woman). These women demonstrate confidence and trust in healthcare providers, considering it natural to undergo genital examinations when necessary for observation or testing. Unlike many who fear such vulnerability, they have no qualms about disrobing: “Many women fear to get there and open their legs, but I am not that kind of women. I am not afraid to take off clothes, I have nothing to hide” Luc, 37 years old, target woman). Furthermore, they exhibit a lack of hesitation in being examined by male healthcare professionals, drawing upon their positive experiences, such as giving birth under the care of a male nurse: “I gave birth with a male nurse and had no problem with that. Why would I be shy to show my genitals for better health?” (Ang, 44 years old, target woman).

While some women, like Dul, may lack proper knowledge about cervical cancer, its transmission, prevention, and treatment options, their trust in healthcare professionals and the information they provide encourages them to willingly participate in screenings. (Dul, 35 years old). For instance, Dul volunteered to be tested after attending a workshop on cervical cancer at a health centre, despite limited understanding of the topic: “1 day I came to the health centre and there was a workshop on cervical cancer. I didn’t understand that much about it, but I volunteered myself to enter the consultancy room to be tested. I remember there was no vinegar and I had to wait a bit. they told me to wait because there was no vinegar or anything. They explained what was going to happen, but after everything they said everything was fine. That was in 2016” (Dul, 35 years old). These women’s faith, often rooted in Christianity, instills fearlessness in undergoing screenings and seeking timely care if needed. They believe that every test result, whether positive or negative, can be faced with prayer and the resolve to continue taking care of oneself: “Every test can have positive or negative result. If it is positive you pray for God and seek for timely treatment. If it is negative, you thank God and mind that you need to keep taking care of yourself. So, there is no reasons to be desperate” (Pau, 30 years old, target woman).

This emphasizes the importance of awareness, breaking societal taboos, and placing a priority on personal health. These women’s attitudes showcase the power of knowledge, trust, and faith in overcoming barriers to screening acceptance and seeking appropriate care.

## Discussion

This study explores how women’s emotional commitments to competing institutional logics impact on the uptake of cervical cancer screening in Mozambique. Emotions play a significant role in shaping individuals’ experiences and decision-making processes. While previous research has shown that individuals embody these logics through their emotions, experiences, and feelings,^19,20,34,35^ our case study in Xai-Xai reveals that women face institutional complexity,^15^ contending with competing logics of material and symbolic rules, as well as socially shared cultural beliefs that shape their choices and decisions.^18,36^ These logics manifest as the logic of preservation and the logic of prevention.

The logic of preservation regards the preservation of social and cultural rules. In the Southern region of Mozambique, the Western Christian civilization and the patriarchal system dominate the patterns of culture, behaviors, the practices of social actors, and influence knowledges, symbols, and expectations^13,37^ that individuals have about themselves and about other members of the society.^35^ These knowledge and beliefs systems are endogenous, unwritten rules, and have local roots that are transmitted gradually from generation to generation.^28,37^ Through socialization and culturalization, individuals absorb schemas of gender inequalities and place male individuals as the natural leaders of the household and rulers of their women.^13^ Although changes have been observed throughout time, within this framework, women are expected to reproduce and safeguard traditional rules, to accept their subordinate position in public matters, to safeguard their reputation, and to preserve their bodies.^29^ The preservation logic is old and endured in Southern Mozambique.

By logic of prevention, we mean universal health interventions like cervical cancer screening. This is grounded on the scientific medicine, expanded since the advent of the modern world. The logic of prevention has been legitimized, accepted and diffused by international health institutions, national health bodies, academic entities, and others.^38^ In this case, the prevention logic for cervical cancer was recommended by the World Health Organization to the Ministry of Health of Mozambique. Women are expected to have enough autonomy to assess and accept elements of this logic and get screened for cervical cancer. The logic of prevention related to cervical cancer screening can be seen as the new logic in Mozambique.

The study reinforces that institutional logics comprise cognitive frames that are highly difficult to change. Despite abundant information and free screening services, cultural bonds and cognitive frames influenced by preservation logic prevent women from being screened for cervical cancer. While both the preservation and prevention logics are accessible and present in the minds of women, its utilization in social interactions is influenced by additional factors. In the study, the majority of women are constrained by the logic of preservation, and this is driven by moral and social emotions. Women adhere to the elements of this logic as they hold emotions like shame in revealing their genitals for observation, fear of undergoing an unfamiliar screening procedure, and subordination to their husbands in decision-making. These emotions bind women to the preservation logic, shaping their individual and group identities and goals. Their values align with societal expectations. Deviating from these expectations carries significant social consequences.

However, in this context, there are also women who have a higher degree of decision-making in their marriages. They gradually internalize and become aware of the benefits of undergoing cervical cancer screening. Despite being part of a social context that upholds the preservation logic, these women gain the ability to assess different logics and progressively integrate elements of the prevention logic into their lives for the sake of their health and family stability. They selectively detach themselves from certain aspects of the preservation logic and begin constructing their identities, values, and choices based not solely on societal expectations, but also on safeguarding their health and well-being. These women reflect on their existing logic and adopt elements of the new prevention logic, leading them to accept cervical cancer screening.

The interplay of constraining and enabling effects of institutional logics explains low screening acceptance. Preservation logic, deeply ingrained in societal norms, creates barriers to screening, reinforced by emotions like shame and fear. On the contrary, autonomy and awareness enable individuals to question prevailing norms, embrace the prevention logic, and overcome detrimental emotions. Positive emotions like autonomy play a crucial role in empowering individuals to detach from prevailing norms and embrace new practices.

The study emphasizes the significance of cultural barriers and the role of emotions in influencing women’s attitudes toward cervical cancer screening. This is valuable insight to the existing literature on barriers to cervical cancer screening,^8-13^ and proposes moving beyond service provision to understanding how women navigate, interpret, and decide to accept or reject screening services. Based on our findings, it can be assumed that increasing women’s autonomy and investing in their empowerment through education would foster greater self-questioning of their home logic and enhance their understanding of the benefits of undergoing screening. This would likely increase their acceptance of cervical cancer screening.

## Conclusions and Implications

This study sheds light on the relevance of women’s emotional commitments to espoused institutional logics in order to understand the reasons behind resistance toward the uptake of cervical cancer screening in Mozambique. It emphasizes the role of emotions and cultural barriers in shaping women’s attitudes toward screening. The findings contribute to the existing literature on barriers to cervical cancer screening by exploring how women navigate and make decisions regarding screening, going beyond the mere provision of services. We adopt an institutional logics approach as it is pertinent to examine the fundamental socially constructed and culturally ingrained cognitive frames and emotional aspects that influence the adoption of cervical cancer screening.

In Xai-Xai, Mozambique, women face a complex interplay of institutional logics. The logic of preservation, driven by social and cultural rules, dominates and influences their choices. Gender inequalities and societal expectations discourage women from undergoing screening. However, some women with higher levels of autonomy gradually integrate elements of the prevention logic into their lives, prioritizing their health and well-being. The low acceptance of screening can be attributed to the constraining and enabling effects of institutional logics. Prevailing norms and values act as barriers, reinforcing adherence to traditional norms and discouraging screening. Emotions play a significant role, either binding women to the preservation logic or enabling them to embrace the new prevention logic. Autonomy and empowerment contribute to detachment from prevailing norms and facilitate acceptance of screening.

Based on these findings, increasing women’s autonomy and empowering them through education is crucial for challenging prevailing norms and enhancing their understanding of screening benefits. Promoting self-questioning of traditional logics and raising awareness about prevention can lead to greater acceptance of screening. The study provides valuable evidence for the Ministry of Health in Mozambique to improve cervical cancer screening uptake. It highlights the importance of improving awareness, empowerment, and women’s education. It also emphasizes the need for cultural awareness among healthcare providers and managers, building trust, engaging communities to integrate cultural norms, and implementing policies that promote gender equality and socio-economic empowerment.

## Supplemental Material

sj-docx-1-his-10.1177_11786329231224619 – Supplemental material for The Influence of Institutional Logics and Emotions on the Uptake of Cervical Cancer Screening: A Case Study From Xai-Xai, Mozambique Supplemental material, sj-docx-1-his-10.1177_11786329231224619 for The Influence of Institutional Logics and Emotions on the Uptake of Cervical Cancer Screening: A Case Study From Xai-Xai, Mozambique by Gefra Fulane, Maria Major, Cesaltina Lorenzoni and Khatia Munguambe in Health Services Insights
