# Supplementary material for: The Influence of Institutional Logics and Emotions on the Uptake of Cervical Cancer Screening: A Case Study From Xai-Xai, Mozambique
Source: Health Serv Insights. 2024 Aug 27;17:11786329231224619. doi: 10.1177/11786329231224619 (PMC11348342; doi:10.1177/11786329231224619)
Supplement: sj-docx-1-his-10.1177_11786329231224619 – Supplemental material for The Influence of Institutional Logics and Emotions on the Uptake of Cervical Cancer Screening: A Case Study From Xai-Xai, Mozambique [file sj-docx-1-his-10.1177_11786329231224619.docx]

**Appendix 1**

*"Creation of Value Prevention Services to Increase Adherence to Cervical Cancer Screening in Mozambique: A Case Study in Gaza"*

**Focus group discussion guide**

Greetings, introduction

- Researcher, assistant, and note takers

**Context and study objectives**

Many women suffer and die from uterine cancer in Mozambique. What many of them don't know is that it is easy to prevent this cancer. There is a way to test and treat when the disease is still at an early stage. But many women only discover this cancer at a very advanced stage, where little can be done. We want to reverse this situation and stop women dying from cancer. For this, your role as women is fundamental in this fight. So this study seeks to understand the knowledge (what women know, what they don't know), the practices and the barriers that women face in getting screened for uterine cancer. The practical part is taking place since May in the Health Centers (individual interviews) and today we are here to understand cancer from the group's perspective. We ask you to collaborate by answering the questions without fear, because the results of the study will serve to improve the uterine cancer prevention program here in the country.

**Focus group discussion process**

The work is simple: we are going to ask you some questions and ask you to answer them. There are no right or wrong answers: all answers are welcome for the improvement of the cervical cancer program. Everything said here, is written here (show informed consent). Here is information about who we are, where we come from, why cancer is of concern, and contacts. It is not written what we are going to talk about here, no. This is confidential. If you agree, I will ask each one of you to sign2 sheets, one stays with us, the other with each one of you.

a) Signing the informed consent and filling out the list of participants

**Perceptions on being a woman, health, and illness**

1.1 What is it to be a woman in the family (wa nsati a ndjanuini)? Go into more detail...

1.2 Is the woman of today the same as the woman of yesterday? Why is that?

1.3. who is the head of the household: man or woman? Why or why not? Go deeper into...

1.4. What does it mean to be well? Nahanha, nini utomi? 1.5 What are the illnesses?

1.5 What are the most common diseases here in Xai-Xai? (list in the box)

1.6. What are the most dangerous diseases? (list from most to least dangerous) Why

do you consider them dangerous?

1.7 How did you learn about these diseases (school, media, family networks

networks, hospitals, etc.)?

**Literacy about reproductive health**
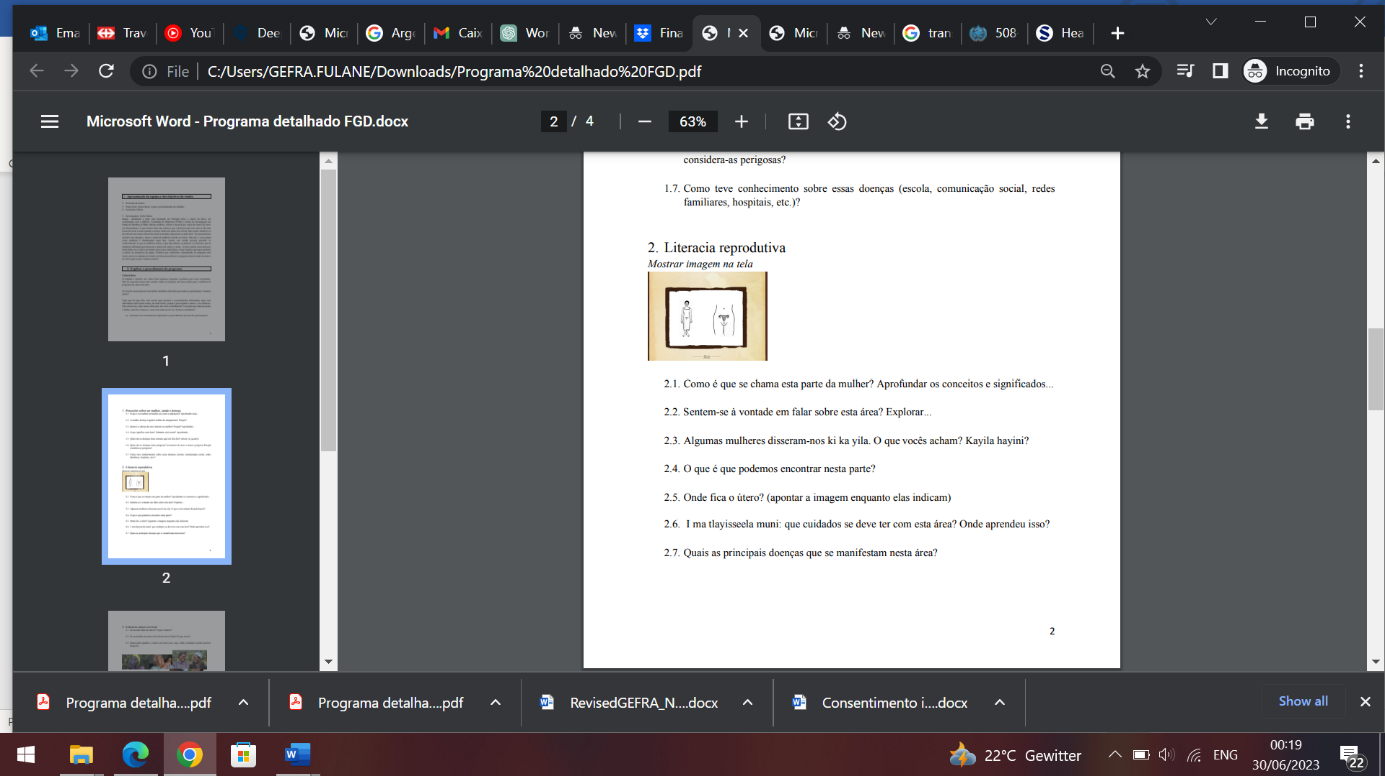


2.1 What is this part of the woman called? Delve into the concepts and meanings...

2.2. do you feel comfortable talking about this area? Explore...

2.3. some women told us ki ka yila. What do you think? Kayila hayini?

2.4. What can we find in this part?

2.5. Where is the uterus? (point to the picture as they point)

2.6. i ma tlayisseela muni: what care should be taken with this area? Where did you learn this?

2.7. What are the main diseases that manifest in this area?

Literacy cervical cancer


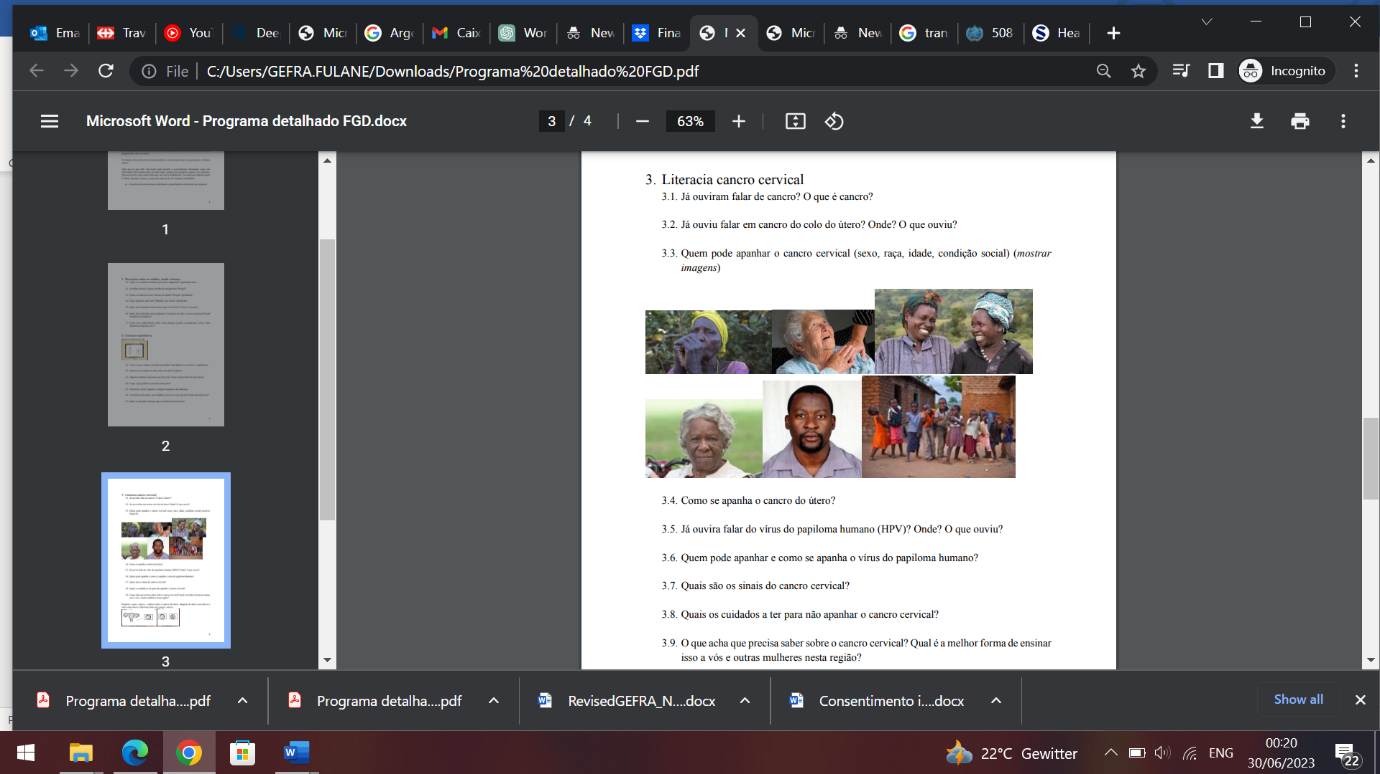
3.1 Have you ever heard of cancer? What is cancer?

3.2. Have you ever heard of cervical cancer? Where? What have you heard?

3.3. Who can get cervical cancer (gender, race, age, social status)

3.4 How do you get uterine cancer?

3.5 Have you ever heard of the human papilloma virus (HPV)? Where? What have you heard?

3.6. who can get and how do you get the human papilloma virus?

3.7 What are the signs of cervical cancer?

3.8. what care should be taken not to get cervical cancer?

3.9. What do you think you need to know about cervical cancer? What is the best way to teach

this to you and other women in this region?

Explain what cancer is, the basics about uterine cancer. Images of a uterus with cancer and

another without cancer. Different stages pre-cancer, cancer...


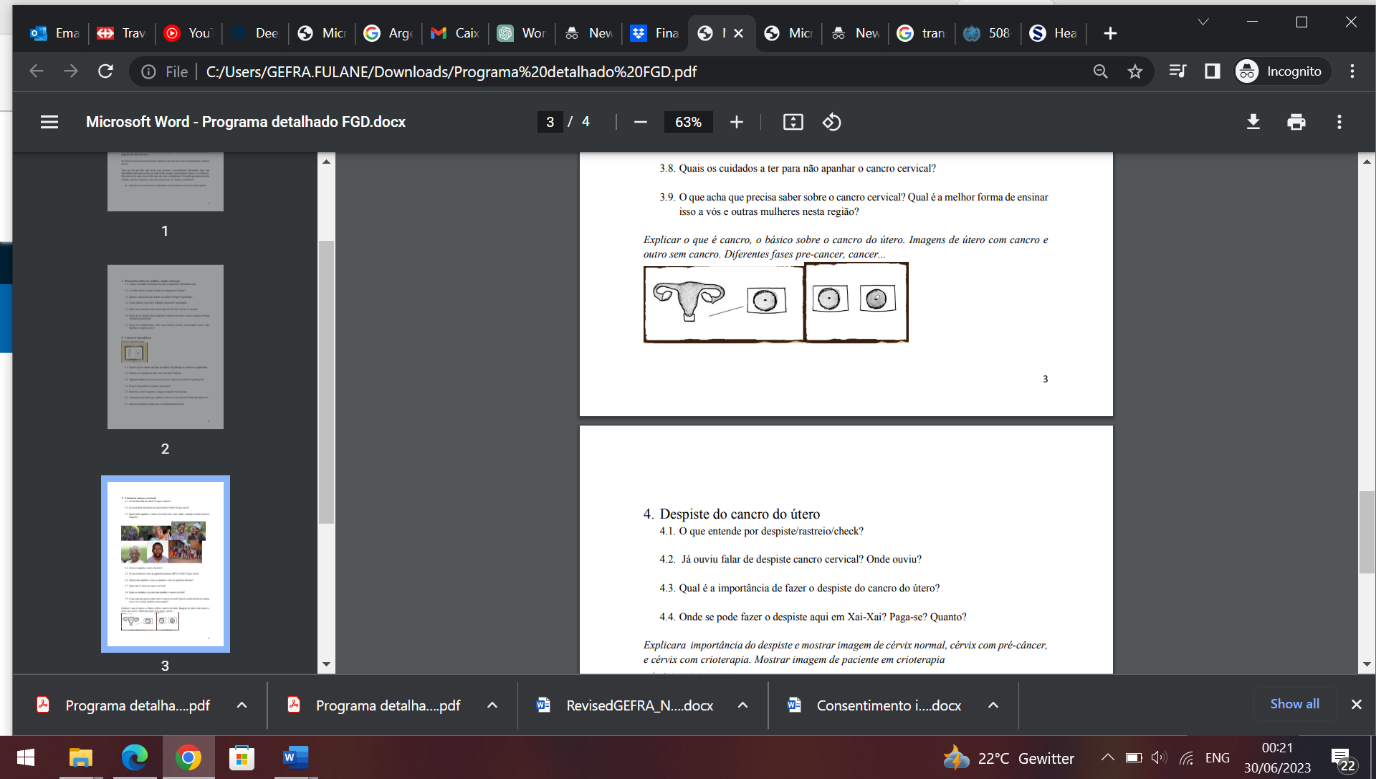


Screening for uterine cancer

4.1. What do you understand by screening/check?

4.2 Have you heard of cervical cancer screening? Where have you heard of it?

4.3 How important is screening for uterine cancer?

4.4. Where can you get screened here in Xai-Xai? Is it paid for? How much does it cost?

Explain the importance of the screening and show an image of a normal cervix, a cervix with pre-cancer and cervix with cryotherapy. Show image of patient in cryotherapy


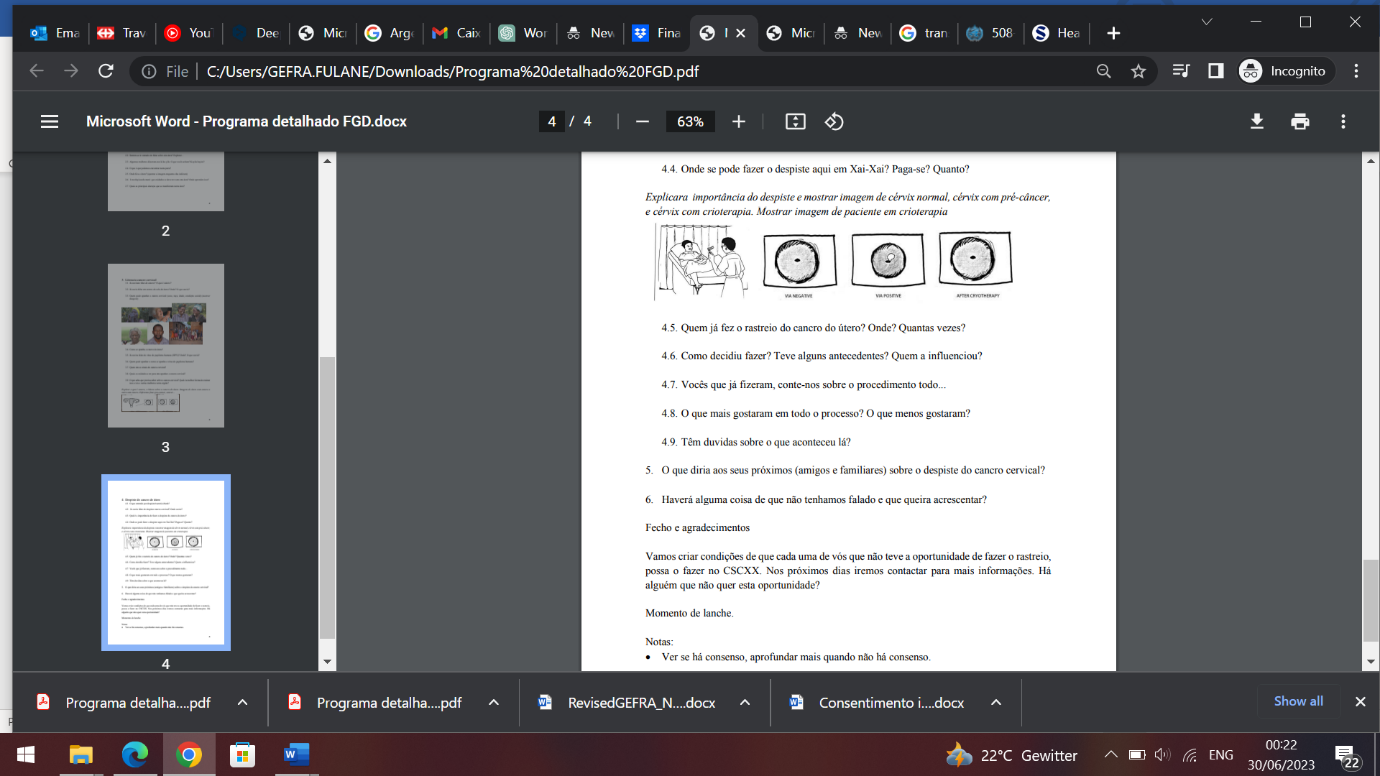


Who has been screened for uterine cancer? Where? how many times?

4.6 How did you decide to have it? Did you have any previous history? Who influenced you?

4.7 If you have already done it, tell us about the whole procedure?

4.8. What did you like most about the whole process? What did you like least?

4.9. Do you have any doubts about what happened there?

5. What would you tell your loved ones (friends and family) about cervical cancer screening?

6. Is there anything we haven't talked about that you would like to add?

*Closing and Acknowledgements*

**Appendix 2**

*"Creation of Value Prevention Services to Increase Adherence to Cervical Cancer Screening in Mozambique: A Case Study in Gaza"*

INFORMATION TO THE PARTICIPANT

AND

INFORMED CONSENT

1. **Researcher: Gefra G. Fulane**
2. **Introduction and background**

Cervical cancer is a global problem, with an estimated 265,672 observed deaths in 2012, nearly 90% of which occur in developing countries. One of the main causes of these deaths is the late screening for precancerous lesions of the cervix. According to the World Health Organization, prevention can prevent up to 80% of deaths and evidence shows that through population-based screening programs, several countries have been able to halt the progression of lesions to cancer. Mozambique, where the prevalence of cervical cancer is between 40 and 96%, has a cervical cancer prevention program, mostly based on screening with low cost techniques such as visual inspection with acetic acid (vinegar). However, due to various reasons only 1% of women between the ages of 30 and 55 are screened annually in the country. Gaza has notable cases of cervical cancer: of the total number of screenings conducted with acetic acid in 2015, 15% were positive (448 de 3077).

This research is part of the PhD course in Tropical Knowledge and Management (Health Sciences) and aims to identify and analyze the barriers that limit women from having cervical cancer screening in Xai-Xai. Guided by Professors Luis Lages and Cesaltina Lorenzoni, we intend with this research to generate scientific knowledge around the coverage and use of screening services and find appropriate recommendations that will serve as object of reflection to improve the screening program strategy.

He/she was selected for the present study which is conducted with the following groups of informants: women over 30, family members (husbands, mothers-in-law, mothers), biomedical health professionals (screening technicians, nurses, doctors), non-biomedical health professionals (zionist pastors and healers), health managers (chiefs, leaders, heads of the screening program).

1. **What participation in the study consists of?**

Participation in the study consists of providing information through interviews and focus group discussions conducted by the principal investigator, which will be recorded and later transcribed. The interview will last an average of 45 minutes and the focus group discussion 1.30 minutes. Overall it seeks to find out:

- Health care seeking and access: geographic, financial, cultural (biomedical and non-biomedical)
- Literacy: reproductive health, chronic diseases, cervical cancer
- Organizational aspects of services
- Perceptions about cervical cancer screening: intimacy, sexual stigma, gender freedom, health rights, cultural acceptance, family and community support networks
- Procedure, specificity of screening, confidentiality, cultural aspects
- Professional-patient relationship

1. **Risks and Benefits**

The present study is expected to bring more benefits than risks for all involved, as the research will gather information about knowledge and practices regarding screening, without invading the privacy and intimacy of the participants, but rather respecting them by clearly explaining the objectives of the study, hiding their real names.

1. **Confidentiality**

Participation in this study implies a guarantee of the highest confidentiality: participants will be guaranteed anonymity, names will be fictitious, and no identifying data will be recorded. The data granted will be for the exclusive use of the research.

1. **Voluntariness**

Participation in the study is voluntary. Participants are free to accept or refuse to participate in the study without any consequences.

1. **Contacts of the investigator in case of questions**

- Address 1: Avenida Samora Machel, Xai-Xai, Mozambique
- Mobile Phone: +258 875754439
- Email: gefragustavo@hotmail.com
- Home Page: https://about.me/gefrafulane
- Institutional Affiliation: Universidade Nova de Lisboa, Campus de Campolide 1099-032, Lisboa Portugal

1. **Declaration of Consent**

Please read the following information carefully. If you feel that something is incorrect or unclear, do not hesitate to request further information. If you agree with the proposal made to you, please sign this document.

Signature of person requesting consent: ... ... ... ... ... ............................................................

... ... ... ... ... ... ... ...... ... ... ... ... ... ... ... ... ... ... ... ... ... ... ... ... ...... ...

I declare that I have read and understood this document and the verbal information given to me by the person/s signing above. I have been assured that I may, at any time, refuse to participate in this study without any consequences. Thus, I agree to participate in this study and allow the use of the data that I voluntarily provide, trusting that they will only be used for this research and the guarantees of confidentiality and anonymity given to me by the researcher.

Name: ... ... ... ... ... ... ...... ... ... ... ...... ... ... ... ... ... ... ... ... ... ... ... ......................

Signature: ... ... ... ... ... ... ... ...... ... ... ... ... ... ... ... ... ... ...... Date: ...... /...... /...........

THIS DOCUMENT CONSISTS OF 2 PAGES AND IS MADE UP IN DUPLICATE: ONE COPY FOR THE RESEARCHER, THE OTHER FOR THE CONSENTING PERSON

**Appendix 3**

*"Creation of Value Prevention Services to Increase Adherence to Cervical Cancer Screening in Mozambique: A Case Study in Gaza"*

**Semi-Structured Interview Protocol**

Researcher: Gefra G. Fulane

Informants: Women > 30 years old

Location: Xai-Xai, Gaza

Sociodemographic and Economic Data

| Residence | Age | Occupation | Marital Status | Education | Religion | Average Income | Who they live with | Number of Children |
| --- | --- | --- | --- | --- | --- | --- | --- | --- |

Daily Routine

- Describe how you generally spend your weekdays and weekends. What are your top priorities? How much free time do you have during the day?

1. Perceptions on being a woman, health, and illness
   1. What is it like to be a woman in the family (ndjango)? And in society? Why? 1.2.
   2. Is today's woman the same as the woman of old? Why is this so?
   3. What are the duties of a woman? And her rights? Why? 1.4.
   4. Who is in charge at home?
   5. If you have to make an important intervention, do you inform/negotiate/ask permission from your husband or other relative? How and why?
   6. What does being there mean to you? Why?
   7. What does not being well mean to you? Why is that?
   8. What does being/being healthy mean to you? Why or why not? 1.9.
   9. What does being sick mean to you? Why do you think so?
   10. What are the most common diseases in the region?
   11. What are the most dangerous diseases that you know? Why do you consider them dangerous?
   12. Which are the diseases that you talk more about with your relatives (friends and family). Why do you consider them dangerous?
   13. How did you learn about these diseases (school, media, family networks, hospitals, etc.)?
   14. Do you feel comfortable talking about the genital anatomy of women? Why? Explain its constitution
   15. What knowledge has been passed on to you about this area? By whom?
   16. What care should a woman take with her intimacy? Where did you learn this? How did you learn it?
   17. What are the main diseases that can affect this area?
2. Health care seeking behaviour
   1. What do you do when you feel you are sick? Why?
   2. When you say religious healing, what do you mean?
   3. When they say prophetic healing, what do they mean?
   4. When you say traditional medicines, what do you mean?
   5. What do your family members do when they are sick? Why?
   6. Are there illnesses that are treated in traditional medicine and others in hospitals? Which ones and why?
3. Access to health care
   1. Geographic
      1. Where is the US? The traditional medicine? The therapeutic church? (time)
      2. Do you think the location of the institutions listed in 3.3 are close or far away?
   2. Financial
      1. How much do you spend to go to the US, TM, CT?
      2. Can you always pay that amount out of pocket?
      3. How much do you pay for the services you look for in the institutions listed in 3.3?
      4. Can you always pay that amount out of pocket?
      5. Are there other associated payments?
   3. Cultural
      1. In what institutions did you use to go to US, MT, CT as a child? Why or why not?
      2. With what group of practitioners do you feel more comfortable? Hivani unga va tolovela?
      3. Do you find these institutions aligned with your culture and tradition? Why do you think so?
      4. What does it take for a person to become US, MT, CP? Do they work differently?
4. Literacy cervical cancer
   1. Have you ever heard of cancer?
   2. What is cancer to you?
   3. Have you ever heard of cervical cancer? Where? What have you heard?
   4. How do you get cervical cancer?
   5. Who can get cervical cancer (men, women, children)?
   6. Have you heard of the human papilloma virus (HPV)? Where? What have you heard?
   7. Who can get and how do you get the human papilloma virus?
   8. What are the signs of cervical cancer?
   9. What care should be taken not to get cervical cancer?
   10. What do you think you need to know about cervical cancer?
   11. What is the best way to teach this?
5. Cervical cancer screening
   1. Screening literacy and perception
      1. What is screening/check for you?
      2. Have you heard of cervical cancer screening? Where did you hear it?
      3. How important is screening for CC?
      4. Where can you get screened here in Xai-Xai? And what additional services can you get at these places? Do you pay? How much?
      5. Do you have any doubts about the cervical cancer screening?
      6. What is the best way to teach you about it?
   2. Experience
      1. Have you ever been screened for cervical cancer? If no, why not and what conditions would be necessary to be screened? (Skip to question 6.18)
      2. If yes, when? Where? How many times?
      3. How did you decide to do it? Did you have any background? Who influenced you?
      4. Did you have any preparation or explanation about the procedure itself and its purpose? What was it? In what language? Did you feel enlightened?
      5. Tell me about the procedure (equipment, installations, privacy, pain, etc)?
      6. Was it with a male or female professional? Did you feel comfortable? Why? Describe the professional: friendly, knowledgeable, etc...?
      7. How would you describe the relationship you had with the professional during the preparation and carrying out of the screening?
      8. Did you tell a relative or a friend that you had the screening? Why did you do it?
      9. Would you like to talk about the result (positive or negative?) If not, how would you evaluate the experience?
      10. Was it worthwhile to be screened? Why? What attitudes and measures have you adopted from then on? (Go to question 6.21/TARV.)
      11. 5Did you have any treatment? Which and where?
      12. Was the treatment on the same day of the screening, same professional? How long did it last? And how many times?
      13. Did you have other treatments for other diseases?
      14. I don't know if this question is appropriate or not... Are you on ART? If not, why and skip to question 7.
      15. If yes, tell us about your experience in managing the two diseases? What care have you received from the hospital?
      16. Some people use MT and others help as religious. And in your case?
      17. What do you think could be improved in the whole process of your experience?
      18. What else would you like to know? What is the best way to teach that?
      19. What for you might get more people to do cc screening?
      20. Is there anything we haven't talked about that you would like to add?

**Appendix 4**

*"Creation of Value Prevention Services to Increase Adherence to Cervical Cancer Screening in Mozambique: A Case Study in Gaza"*

**Semi-Structured Interview Protocol**

Researcher: Gefra G. Fulane

Informants: health professionals

Location: Xai-Xai, Gaza

Sociodemographic and Economic Data

| Residence | Age | Occupation | Marital Status | Education | Religion | Average Income | Who they live with | Number of Children |
| --- | --- | --- | --- | --- | --- | --- | --- | --- |

1. Perceptions, demand and access to services
   1. What is it like to be a woman in your society and family?
   2. What are the duties and rights of a woman?
   3. Who in your household has more influence on decisions about your body, your health, and the choices you make? Why? 1.4.
   4. What are the most common diseases in the region?
   5. What are the most dangerous diseases that you know of? Why do you consider them dangerous?
   6. How should you treat/attend a patient? Why?
2. Literacy cervical cancer
   1. Explain what you understand by cancer
   2. What about cervical cancer? How does it develop? Who can get it (men, women, children)?
   3. What are the signs of cervical cancer? What precautions should be taken to avoid developing cervical cancer?
   4. What is the human papilloma virus? How does it develop? Who can contract HPV (men, women, children)? What are the signs of HPV?
   5. What care should be taken not to contract HPV?
3. Screening for cervical cancer
   1. How long have you been working with CC screening?
   2. What tests do you use for CC screening?
   3. Have you had any specific training? Which one? When?
   4. Do you imagine you could be here doing the screening sometime in your life at this time and age? Please explain.
   5. What information do you think is important to obtain from the client before the screening? Have you obtained this information? How?
   6. What information do you think is important to give to the client before the screening? Have you given that information? How?
   7. Is there any preparation or pelvic examination before the screening? How do you do it?
   8. Is the way you manage HV+ clients different from HIV- clients? Please explain.
   9. Describe your clients' profile: shy, distrustful, embarrassed, comfortable, literate, Portuguese-speaking, have notions of reproductive health, have notions of what cancer is?
   10. What do you think could be improved in the users' profile?
   11. Have you exchanged information about the clients with other professionals in other sections? How (oral, written, reports)?
   12. How do you follow up clients after the screening?
   13. Have you ever done a HS screening? If not, why? How often and when? Can you tell me about the experience: gender of the professional, comfort, pain, privacy?

1. What do you think could be improved in the whole process of your experience (organization of services, adherence of users, follow-up, incentives, etc.)?

5. Is there anything that we have not talked about that you would like to add?

**Appendix 5**

*"Creation of Value Prevention Services to Increase Adherence to Cervical Cancer Screening in Mozambique: A Case Study in Gaza"*

**Semi-Structured Interview Protocol**

Researcher: Gefra G. Fulane

Informants: family members (mothers and mothers in law)

Location: Xai-Xai, Gaza

Sociodemographic and Economic Data

| Residence | Age | Occupation | Marital Status | Education | Religion | Average Income | Who they live with | Number of Children |
| --- | --- | --- | --- | --- | --- | --- | --- | --- |

1. Perceptions about health and illness

1.1 What does well-being mean to you? Why do you think so?

1.2 What does being healthy mean to you? Why or why not? 1.3.

1.3 What does being sick mean to you? Why? 1.4.

1.4. what are the most common diseases in the region?

1.5. what are the most dangerous diseases that you know? Why do you consider them dangerous?

1.6. How did you learn about these diseases (school, media, family networks, hospitals, etc.)

2. Perceptions about being a woman

2.1. What is it to be a woman in your society and family?

2.2 What are the duties and rights of a woman?

2.3. Who in your household has more influence over decisions about your body from your wife/daughter/son-in-law? Why?

2.4 If she has to make an important intervention, who do you inform/negotiate/ask permission from? How and why?

2.5 What care should a woman take with her intimacy?

2.6 What are the main diseases that can affect this area?

2.7 What teachings do you give to the younger generations (daughters, nieces, etc.)?

3. Health care demand

3.1. What do you do when you feel you are sick? Why? 3.2.

3.2 What do your family members do when they are sick? Why? 3.3.

3.3. do you seek the same kind of help/solution for all illnesses? Explain the reasons.

3.4. Do your family members seek the same kind of help/solution when they are sick?

4. Access to health care

a) Geographic

4.1. Where are the institutions answered in question 3.3 located (list time and distance to each)?

4.2. Do you think that the location of the institutions listed in 3.3 are close or distant?

b) Financial

4.3 How much do you spend to go to the institutions listed in question 3.3?

4.4 Can you always pay that amount out of pocket?

4.5 How much do you pay for the services you seek in the institutions listed in 3.3?

4.6 Can you always pay that amount out of pocket?

4.7 Are there other associated payments?

c) Cultural

4.8. Which institutions in 3.3 did you use to go to as a child? Why?

4.9. Do you think these institutions are aligned with your culture and tradition? Why do you think so?

4.10. Do you know and understand the organization and operation of these institutions?

4.11. Do you have autonomy and feel at ease in dialoguing with the professionals at these institutions?

5. Cervical cancer literacy

5.1 Have you ever heard of cancer? Where? What have you heard?

5.2. What is cancer to you?

5.3. Have you ever heard of cervical cancer? Where? What have you heard?

5.4. Who can get cervical cancer (men, women, children)?

5.5. how do you get cervical cancer?

5.6. Have you heard of the human papilloma virus? Where? What have you heard?

5.7 How do you get the human papilloma virus?

5.8 What are the signs of cervical cancer?

5.9 What care should be taken not to get cervical cancer?

6. Cervical cancer screening

a) Screening literacy and perception

6.1 What is screening for you?

6.2 Have you ever heard of cervical cancer screening? Where did you hear it?

6.3 What have you heard and how often?

6.4 How important is it to get screened?

6.5. Where can you get screened here in Xai-Xai? Is it paid for? How much is it?

b) Experience

6.6 Has your daughter/daughter-in-law ever been screened for cervical cancer? If not, why not? Does screening imply.... is it peaceful for you for your daughter/daughter-in-law to have it done? What conditions would be necessary to be screened? (Skip to question 7.)

6.7. If yes, how did you become aware of it? And how did it all start? What did you tell him and how did you tell him?

6.8. Who did you go with and how did it all go at the hospital?

6.9. Tell me about the outcome, if it was worth it, etc.

6.10. Was the treatment on the same day of the screening, same professional? How long did it last? And how many times?

6.11. What do you think could be improved in the whole process of your experience?

7. What would you tell your loved ones (friends and family) about cervical cancer screening?

8. Is there anything we haven't talked about that you would like to add?
